# Trends in prognosis and use of SGLT2i and GLP-1 RA in patients with diabetes and coronary artery disease

**DOI:** 10.1186/s12933-024-02365-1

**Published:** 2024-08-07

**Authors:** Viveca Ritsinger, Kamila Avander, Bo Lagerqvist, Pia Lundman, Anna Norhammar

**Affiliations:** 1 Cardiology Unit, Department of Medicine K2, Karolinska Institutet, Stockholm, Sweden; 2 Department of Research and Development, Region Kronoberg, Växjö, Sweden; 3 Division of Cardiovascular Medicine, Department of Clinical Sciences, Karolinska Institutet, Danderyd Hospital, Stockholm, Sweden; 4 Department of Medical Sciences, Cardiology and Uppsala Clinical Research Center, Uppsala University, Uppsala, Sweden; 5 Capio S:t Görans Hospital, Stockholm, Sweden

## Abstract

**Objective:**

To explore trends in prognosis and use of glucose-lowering drugs (GLD) in patients with diabetes and coronary artery disease (CAD).

**Research design and methods:**

All patients with diabetes and CAD undergoing a coronary angiography between 2010 and 2021 according to the Swedish Angiography and Angioplasty Registry were included. Information on GLD (dispended 6 months before or after coronary angiography) was collected from the Swedish Prescribed Drug Registry. Data on major cardiovascular events (MACE; mortality, myocardial infarction, stroke, heart failure) through December 2021 were obtained from national registries. Cox proportional survival analysis was used to assess outcomes where cardioprotective GLD (any of Sodium Glucose Lowering Transport 2 receptor inhibitors [SGLT2i] and Glucagon Like Peptide Receptor Agonists [GLP-1 RA]) served as a reference.

**Results:**

Among all patients (*n* = 38,671), 31% had stable CAD, and 69% suffered an acute myocardial infarction. Mean age was 69 years, 67% were male, and 81% were on GLD. The use of cardioprotective GLD increased rapidly in recent years (2016–2021; 7–47%) and was more common in younger patients (66 vs. 68 years) and men (72.9% vs. 67.1%) than other GLD. Furthermore, compared with other GLD, the use of cardioprotective GLD was more common in patients with a less frequent history of heart failure (5.0% vs. 6.8%), myocardial infarction (7.7% vs. 10.5%) and chronic kidney disease (3.7% vs. 5.2%). The adjusted hazard ratio (HR) (95% CI) for MACE was greater in patients on other GLD than in those on cardioprotective GLD (1.10; 1.03–1.17, *p* = 0.004). Trend analyses for the years 2010–2019 revealed improved one-year MACE in patients with diabetes and CAD (year 2019 vs. 2010; 0.90; 0.81-1.00, *p* = 0.045), while 1-year mortality was unchanged.

**Conclusions:**

The prescription pattern of diabetes medication is changing quickly in patients with diabetes and CAD; however, there are worrying signals of inefficient use prioritizing cardioprotective GLD to younger and healthier individuals at lower cardiovascular risk. Despite this, there are improving trends in 1-year morbidity.

**Supplementary Information:**

The online version contains supplementary material available at 10.1186/s12933-024-02365-1.

## Introduction

Diabetes is a strong risk factor for the development of cardiovascular disease (CVD), which increases the risk by 2–3 times [1, 2]. Additionally, there is an increased risk of complications after an acute cardiovascular (CV) event [3–5]. Despite improved management of acute coronary syndrome during recent decades, longevity after a CV event is still compromised in patients with diabetes compared to those without diabetes [6]. Novel cardioprotective glucose-lowering drugs ([GLD], i.e., Glucagon Like Peptide Receptor Agonists [GLP-1 RA] and Sodium Glucose Lowering Transport 2 receptor inhibitors [SGLT2i]) have shown impressive CV preventive effects in high-risk patients with type 2 diabetes [7–9], with the greatest absolute benefit in those at high risk [10] emphasizing the importance of prescribing novel GLD in those patients [11, 12]. In addition, according to the latest international guidelines on diabetes and CVD, GLP-1 RA and SGLT2i should be prioritized in patients with established CVD independent of glucose control or metformin use [11, 12]. Novel cardioprotective GLD was introduced in Europe a decade ago; SGLT2i was introduced in 2012, and GLP-1 RA was introduced in 2013. However, there are limited data on the actual prescription pattern of novel GLD and whether patients with type 2 diabetes at high risk for CV events are prescribed these drugs in a modern real-life setting. Therefore, the aim of this study was to explore the trends and real-life use of novel GLD in patients with diabetes and coronary artery disease (CAD) from a national perspective over recent decades. Furthermore, we aimed to analyze the characteristics of patients receiving novel cardioprotective GLD. Finally, we explored trends in outcomes in patients with diabetes and CAD.

## Methods

### Data sources and patient cohort

All patients with diabetes who underwent coronary angiography in Sweden with the indication stable CAD, non-ST-segment elevation myocardial infarction (NSTEMI) or ST-segment elevation myocardial infarction (STEMI) between 2010 and 2021, as reported in the Swedish Angiography and Angioplasty Registry (SCAAR) were included in the study. SCAAR includes verified information on every patient undergoing coronary angiography at all centers performing coronary angiography (*n* = 30), and the Swedish Register of Cardiac Intensive Care (RIKS-HIA) contains monitored information from all cardiac intensive care units in Sweden (*n* = 78). Both registries (RIKS-HIA and SCAAR) are part of the national SWEDEHEART registry [13]. Information on diabetes diagnosis was obtained from the SCAAR and the National Patient Registry (NPR; International Classification of Diseases [ICD]-10 codes E10-E14). Information on the dispensed GLD was derived from the Swedish Prescribed Drug Registry (PDR; ATC-code A10) from six months before to six months after the index coronary angiography to understand whether treatment with novel cardioprotective GLD (GLP-1 RA and SGLT2i) at the time of coronary angiography affects outcomes. National registries were used to follow patients for major CV events until December 31, 2021, and all-cause death until March 31, 2022 (a difference in follow-up time due to delay in the possibility of deriving data from the NPR). To obtain information on mortality and comorbidities during follow-up, the SWEDEHEART data were cross-referenced with the Swedish Population Register and the NPR. Mortality during the first 30 days was excluded from the analysis to limit healthy selection bias of dispensed GLD. Mortality cause by year after the index coronary angiography and during the first year after coronary angiography each year from 2010 to 2021 was analyzed. Mortality causes were classified according to the ICD-10 codes, as set by the physician in charge, and were categorized into thirteen classes of main mortality causes.

### Definitions

Diabetes mellitus at the index coronary angiography was defined as a diabetes diagnosis either registered in the SCAAR or in the NPR (ICD-code E10- E11). Patients were not categorized by type of diabetes.

Glucose-lowering therapies and treatments six months before or after coronary angiography were classified into the following dispended groups of medication during this time period and based on registered ATC codes in the Prescribed Drug Register (SGLT2i: ATC code A10BK, GLP-1 RA: ATC code A10BJ):

I. *Any of SGLT2i or GLP-1 RA* (with or without other GLD).
II. *SGLT2i* (with or without other GLD but not GLP-1 RA).
III. *GLP-1 RA* (with or without other GLD but not SGLT2i).
IV. *SGLT2i and GLP-1 RA* (use of both with and without other GLD).
V. *Other GLD* (without SGLT2i or GLP-1 RA).
VI. *No GLD* (but diabetes diagnosis).

### Outcome

Major adverse cardiovascular event (MACE): first of all-cause death, MI(ICD-10 code I21-I22), stroke (ICD-10 code I63) or heart failure (ICD-10 code I50) through December 31, 2021.

All-cause death was collected until March 31, 2022.

### Statistical analysis

Baseline characteristics and outcomes were compared across the GLD classes and are presented as the mean and standard deviation (SD) for continuous variables and as numbers and percentages for categorical variables. SGLT2i and GLP-1 RA were analyzed either as monotherapy (only SGLT2i or only GLP-1 RA) or in combination; either one of them (any SGLT2i or GLP-1 RA) or both (SGLT2i and GLP-1 RA). To compare baseline characteristics between the different groups, the χ^2^ test or Fisher’s exact test was used. In addition, a sensitivity analysis limited to patients included between 2020 and 2021 was performed. Cumulative event rates for the specified outcomes were calculated using the Kaplan‒Meier method and are graphically displayed. Outcomes associated with different GLD groups (where the group Any of SGLT2i or GLP-1 RA served as a reference) were analyzed in adjusted Cox proportional hazards models (HR, 95% confidence interval) and were adjusted for age, sex, smoking, previous diagnosis of MI/ heart failure/ cancer/ hypertension/ hyperlipidemia/ renal failure/ stroke/ peripheral artery disease, year of inclusion, indication, and angiographic findings. A two-sided p value of < 0.05 was considered to indicate statistical significance. All analyses were conducted using the R statistical program (version 4.2.2). Time trends in one-year outcomes were assessed, with the year 2010 serving as a reference.

### Ethical consideration

The study was approved by the Swedish Ethical Review Authority (Dnr 2012/60 − 31/2, 2017/432 − 32, 2020–04252) and was conducted in accordance with the Declaration of Helsinki. No individual patient consent for entering the SWEDEHEART registry was obtained, but patients were informed about permission to opt out.

## Results

### Baseline characteristics

Baseline characteristics are depicted in Table 1. In the total cohort (*n* = 38 671 with diabetes), 81.1% were on any GLD (*n* = 31 368), the mean age was 68.6 (SD ± 10.6) years, and 67.3% (*n* = 26 017) were male. The indications for coronary angiography were stable CAD in 31% and acute MI in 69% (STEMI 40%, NSTEMI 60%) of the study population. The majority of those with MI were treated with PCI (Table 1).

**Table 1:** Patient characteristics at baseline, mean (SD) or n (%) stratified by glucose-lowering drugs (GLD)

	SGLT2i (*n* = 3191)	GLP1-RA (*n* = 1570)	SGLT2i + GLP1-RA (*n* = 640)	Other GLD (*n* = 25 967)	No GLD (*n* = 7303)	*p* value 1	Any SGLT2i/GLP1-RA (*n* = 5401)	*p* value 2 Any SGLT2i/GLP1-RA vs. Other GLD
Age (years) (mean, SD)	66.2 (10.2)	65.2 (9.8)	63.7 (9.3)	68.4 (10.4)	71.8 (10.8)	< 0.001	65.6 (10.0)	< 0.001
Sex (female) (n, %)	783 (24.5)	513 (32.7)	167 (26.1)	8540 (32.9)	2651 (36.3)	< 0.001	1463 (27.1)	< 0.001
Current smoker	599 (18.8)	233 (14.8)	132 (20.6)	4363 (16.8)	1152 (15.8)	< 0.001	964 (17.8)	0.016
BMI (kg/m^2^)	29.3 (5.1)	32.3 (5.2)	31.3 (5.4)	29.0 (5.0)	28.1 (5.3)	< 0.001	30.4 (5.3)	< 0.001
Weight (kg)	88.2 (17.3)	97.0 (17.8)	94.6 (18.2)	85.9 (16.9)	82.3 (17.0)	< 0.001	91.5 (18.0)	< 0.001
Left Ventricular Ejection fraction (LVEF %**)**						< 0.001		0.087
LVEF ≥ 50%	1265 (54.7)	545 (62.1)	240 (56.5)	9107 (58.5)	2293 (48.5)		2050 (56.7)	
LVEF 40–49%	575 (24.8)	195 (22.2)	106 (24.9)	3481 (22.4)	969 (20.5)		876 (24.2)	
LVEF 30–39%	314 (13.6)	105 (12.0)	64 (15.1)	2124 (13.6)	740 (15.6)		483 (13.4)	
LVEF < 30%	160 (6.9)	32 (3.6)	15 (3.5)	858 (5.5)	656 (13.9)		207 (5.7)	
Blood glucose (mmol/L)	12.2 (5.1)	12.5 (5.0)	12.7 (5.2)	11.9 (5.2)	10.4 (5.3)	< 0.001	12.3 (5.1)	< 0.001
CRP (mg/L)	18.0 (41.7)	18.4 (40.2)	15.7 (39.1)	17.9 (39.9)	24.3 (47.9)	< 0.001	17.8 (41.0)	0.924
Creatinine (mmol/L)	79.5 (27.2)	89.8 (45.6)	82.2 (42.8)	92.0 (64.2)	107.1 (90.2)	< 0.001	82.7 (35.7)	< 0.001
Previous disease								
Heart failure	139 (4.4)	100 (6.4)	25 (3.9)	1770 (6.8)	700 (9.6)	< 0.001	264 (4.9)	< 0.001
PAD	104 (3.3)	70 (4.5)	26 (4.1)	1341 (5.2)	535 (7.3)	< 0.001	200 (3.7)	< 0.001
MI	245 (7.8)	122 (7.9)	43 (6.8)	2736 (10.8)	890 (12.2)		410 (7.7)	< 0.001
Stroke	197 (6.2)	137 (8.7)	46 (7.2)	2167 (8.3)	822 (11.3)	< 0.001	380 (7.0)	0.001
Renal failure	71 (2.2)	104 (6.6)	22 (3.4)	1357 (5.2)	617 (8.4)	< 0.001	197 (3.6)	< 0.001
Cancer	81 (2.5)	41 (2.6)	13 (2.0)	957 (3.7)	374 (5.1)	< 0.001	135 (2.5)	< 0.001
Dementia	9 (0.3)	0 (0)	1 (0.2)	79 (0.3)	50 (0.7)	0.002	10 (0.2)	0.175
Hypertension	2332 (73.7)	1297 (83.0)	522 (82.6)	19 938 (77.6)	5388 (73.8)	< 0.001	4151 (77.5)	0.815
Hyperlipidemia	1760 (56.1)	1036 (66.8)	390 (61.9)	15 782 (61.8)	3520 (48.2)	< 0.001	3186 (59.9)	0.009
Indication						< 0.001		< 0.001
Stable CAD	772 (24.2)	616 (39.2)	191 (29.8)	8725 (33.6)	1683 (23.0)		1579 (29.2)	
NSTEMI	¨1366 (42.8)	627 (39.9)	245 (38.3)	11 253 (43.3)	2482 (34.0)		2238 (41.4)	
STEMI	1053 (33.0)	327 (20.8)	204 (31.9)	5989 (23.1)	3138 (43.0)		1584 (29.3)	
Angiographic findings						< 0.001		< 0.001
Normal	309 (9.7)	275 (17.5)	75 (11.7)	3425 (13.2)	935 (12.8)		659 (12.2)	
1-vessel	1080 (33.8)	513 (32.7)	239 (37.3)	7961 (30.7)	2230 (30.5)		1832 (33.9)	
2-vessel	774 (24.3)	365 (23.2)	147 (23.0)	6282 (24.2)	1742 (23.9)		1286 (23.8)	
3-vessel	772 (24.2)	311 (19.8)	141 (22.0)	6150 (23.7)	1601 (21.9)		1224 (22.7)	
Left main	254 (8.0)	100 (6.4)	37 (5.8)	2111 (8.1)	774 (10.6)		391 (7.2)	
Revascularization method								
PCI	2344 (73.5)	1040 (66.2)	486 (75.9)	16 819 (64.8)	4993 (68.4)	< 0.001	3870 (71.7)	< 0.001
CABG within 3 months after index angiography	182 (5.7)	92 (5.9)	32 (5.0)	1661 (6.4)	253 (3.5)	< 0.001	306 (5.7)	0.047
Stent during PCI (n)	1.4 (1.1)	1.3 (1.0)	1.4 (1.0)	1.4 (1.1)	1.4 (1.1)	< 0.001	1.4 (1.1)	0.481
Follow-up time (days)	811.8 (600.8)	1482.4 (1071.8)	713.4 (555.2)	2395.1 (1188.5)	2360.9 (1209.9)	< 0.001	995.6 (825.5)	< 0.001
Medications within 6 months to index angiography								
Insulin	1108 (34.7)	905 (57.6)	339 (53.0)	12 395 (47.7)	0 (0)	< 0.001	2352 (43.5)	< 0.001
Metformin	2389 (74.9)	1019 (64.9)	471 (73.6)	17 152 (66.1)	0 (0)	< 0.001	3879 (71.8)	< 0.001
Sulfonylurea	249 (7.8)	152 (9.7)	45 (7.0)	3286 (12.7)	0 (0)	< 0.001	446 (8.3)	< 0.001
SGLT2i	3191 (100)	0 (0)	640 (100)	0 (0)	0 (0)	< 0.001	3831 (70.9)	< 0.001
DPP4i	519 (16.3)	123 (7.8)	56 (8.8)	2804 (10.8)	0 (0)	< 0.001	698 (12.9)	< 0.001
GLP-1 RA	0 (0)	1570 (100)	640 (100)	0 (0)	0 (0)	< 0.001	2210 (40.9)	< 0.001
Any diabetes medication prior admission	2648 (83.0)	1500 (95.5)	606 (94.7)	23 249 (89.5)	2602 (35.6)	< 0.001	4754 (88.0)	0.001

*SGLT2i* Sodium Glucose Lowering Transport 2 receptor inhibitors, *GLP-1 RA* Glucagon Like Peptide Receptor Agonists, *BMI* body mass index. CRP: C-reactive protein, *PAD* peripheral artery disease, *MI* myocardial infarction, *CAD* Coronary artery disease, *NSTEMI* non-ST-segment elevation myocardial infarction, *STEMI* ST-segment elevation myocardial infarction, *PCI* percutaneous coronary intervention, *CABG* coronary artery bypass graft, *DPP4i* Dipeptidyl peptidase 4 inhibitors

### Trends in the prescription pattern of GLD

**Fig. 1:**
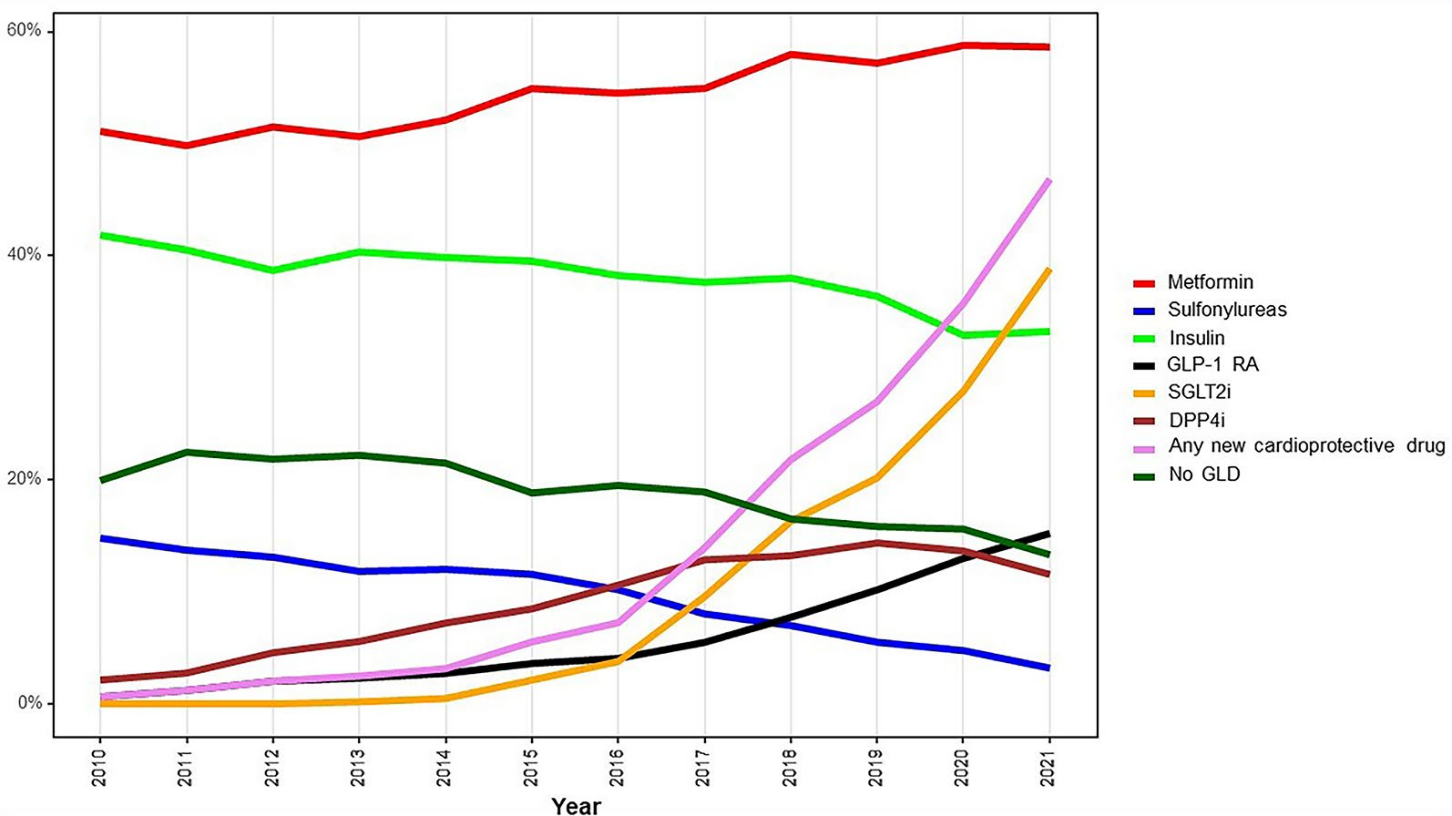
Time trends in the use of glucose-lowering drugs in patients with coronary artery disease in Sweden between 2010 and 2021. *GLP-1 RA* Glucagon Like Peptide Receptor Agonists, *SGLT2i* Sodium Glucose Lowering Transport 2 receptor inhibitors, *DPP4i* Dipeptidyl peptidase 4 inhibitors, *GLD* Glucose-lowering drugs. Any new cardioprotective drug = any of SGLT2i or GLP-1 RA (with or without other GLD)

The proportions of GLD used over time are depicted in Fig. 1. The GLD landscape changed during the years 2010–2021 with increasing use of any of SGLT2i or GLP-1 RA, and the proportion increased from 7% in 2016 to 47% in 2021, which was mainly driven by the use of SGLT2i (from 4 to 38%) and less of GLP-1 RA (4–15%). The use of metformin increased slightly (from 51% in 2010 to 59% in 2021). The use of insulin (42% in 2010 to 33% in 2021) and the absence of glucose-lowering treatment (20% in 2010 to 13% 2021) decreased slightly, and the use of sulfonylurea decreased dramatically (15–3% 2021). The proportion of GLD-naïve patients before the index event was highest in the SGLT2i group (17% compared to 4% in the GLP-1 RA group), as depicted in Supplemental Fig. 1 (see Table 1 for details).

### Characteristics of patients who received the novel GLD vs. those who received other GLD

Patients on any of SGLT2i or GLP-1 RA (*n* = 5401) compared to other GLD (*n* = 25 967) were younger (65.6 vs. 68.4 years; *p* < 0.001), were less likely to be women (27.1% vs. 32.9%; *p* < 0.001) and had less often other comorbidities as previous MI (7.7% vs. 10.8%; *p* < 0.001), previous heart failure (4.9% vs. 6.8%; *p* < 0.001), peripheral artery disease (3.7% vs. 5.2%; *p* < 0.001) and renal failure (3.6% vs. 5.2%; *p* < 0.001). The same pattern was observed in a subgroup analysis of patients included between 2020 and 2021 (Supplemental Table 1). Patients who were prescribed the combination of SGLT2i and GLP-1 RA (*n* = 640) vs. any of the other GLD groups were the youngest with more hypertension, while there were only marginal differences in other comorbidities.

### Characteristics of patients prescribed SGLT2i vs. GLP-1 RA

Men (75.5% vs. 67.3%) with a lower BMI (29 vs. 32 kg/m^2^) and creatinine (80 vs. 90 mmol/L) were more likely to be prescribed SGLT2i (*n* = 3191) than were those prescribed GLP-1 RA (*n* = 1570); additionally, these patients were less likely to have hypertension (73.1% vs. 82.6%) and hyperlipidemia (55.2% vs. 66.0%) and were less likely to have been previously diagnosed with heart failure (4.4% vs. 6.4%), renal failure (2.2% vs. 6.6%) or stroke (6.2% vs. 8.7%).

### Characteristics of patients without any GLD

The group of patients without any GLD were the oldest, were more often female, had the highest rate of comorbidity and had the highest proportion of LVEF < 30% at discharge.

### Outcome

The mean total follow-up time was 6.0 years (min–max 17 to 4399 days), 2.7 (SD 2.3) years for patients on any of SGLT2i or GLP-1 RA and 6.6 (SD 3.3) years for patients on other GLD.

### Trends in overall outcome in the years 2010–2021

**Fig. 2:**
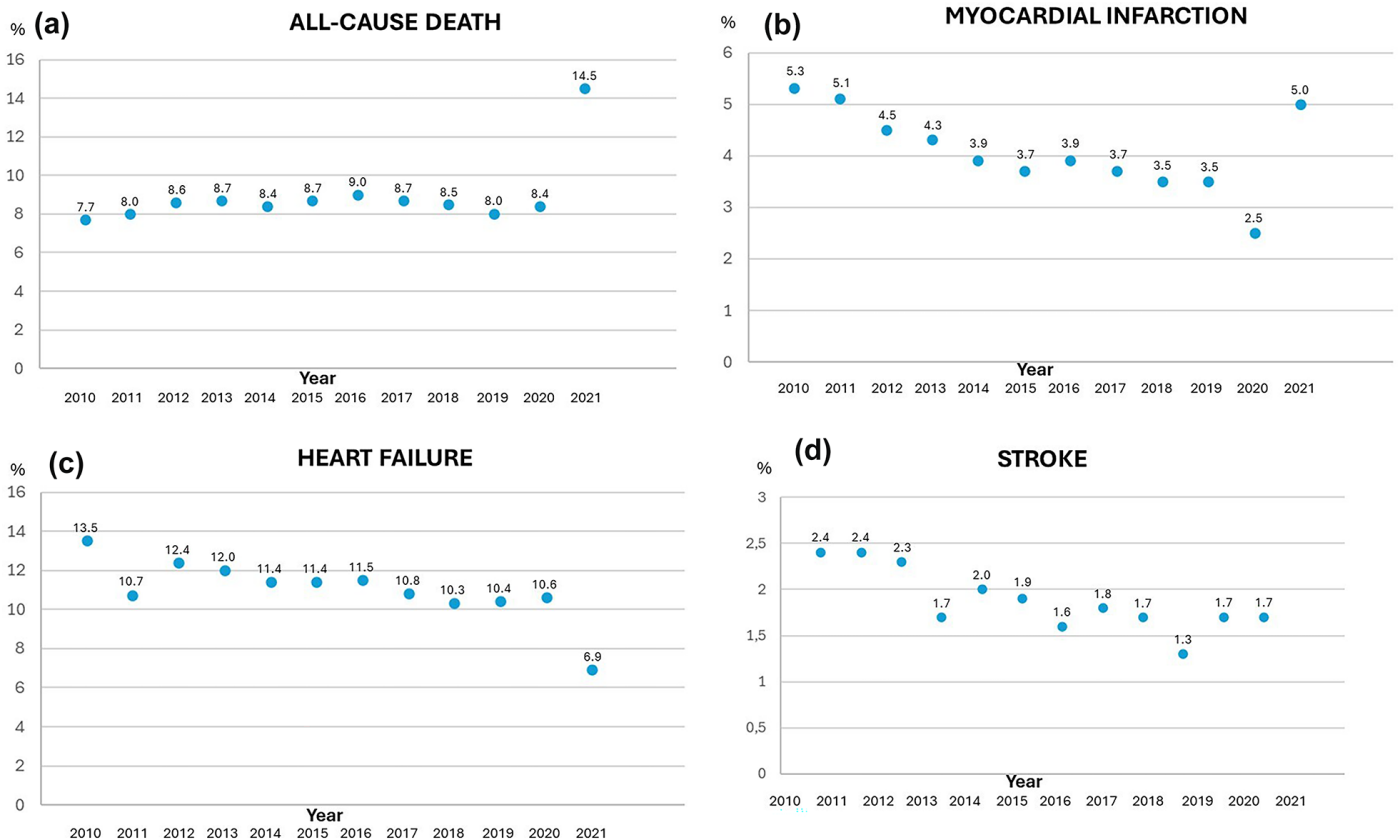
One-year event rates of **a** all-cause death, **b** myocardial infarction, **c** stroke and **d** heart failure by year between 2010 and 2021

**Fig. 3:**
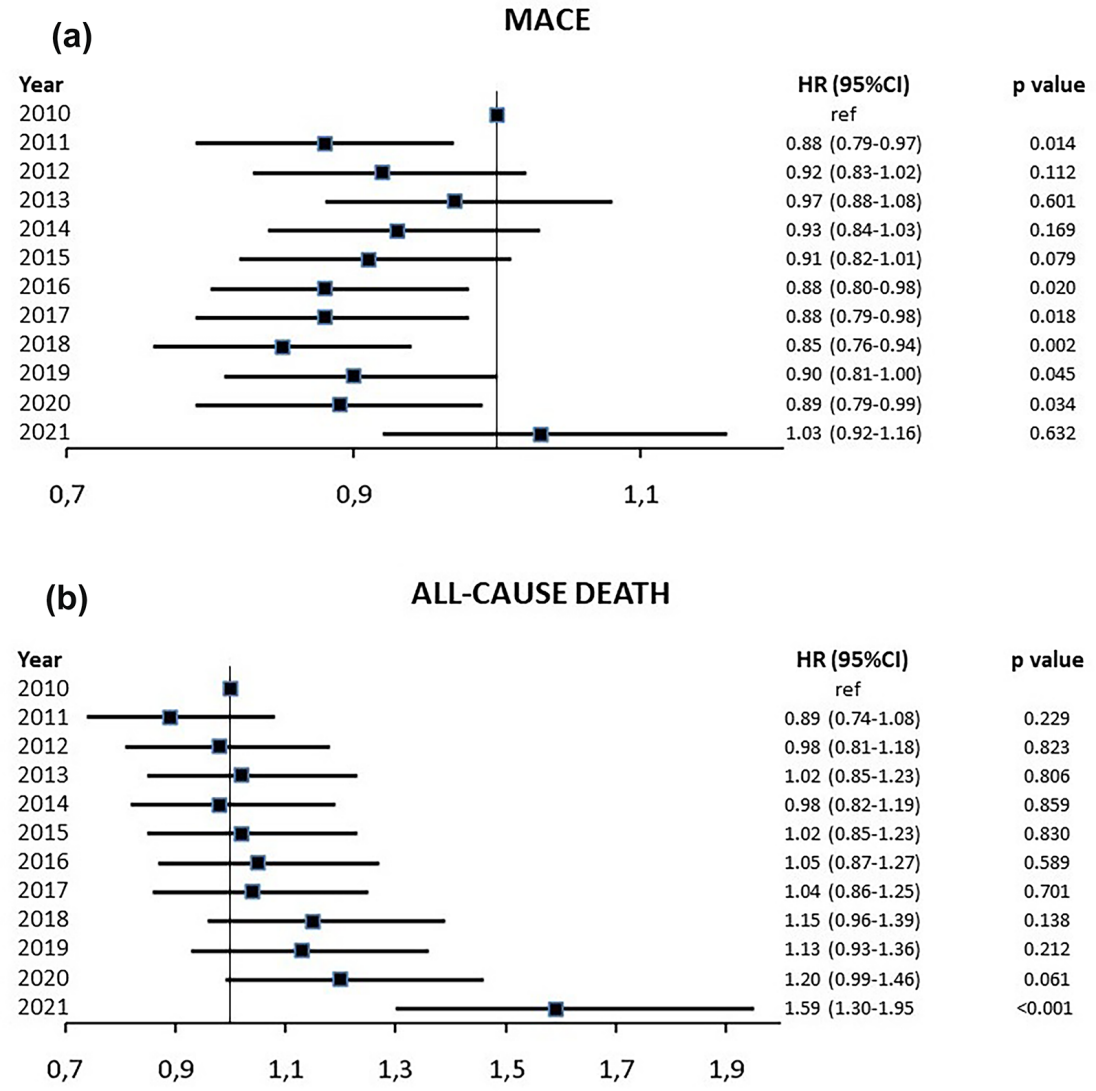
Adjusted associated HR (95% CI) for **a** MACE (all-cause death, myocardial infarction, stroke or heart failure) and **b** all-cause death by year between 2010 and 2021. The year 2010 served as a reference, with an HR of 1.0. Year 2020 and 2021 should be interpreted with caution given the COVID-19 pandemic

When analyzing trends in one-year outcomes (with 2010 as a reference), there was a decrease in the absolute event rate of MACE from 26.0% in 2010 to 22.7% in 2019 (excluding 2020–2021 due to the COVID-19 pandemic). The absolute yearly event rates of the different components of MACE (all-cause death, myocardial infarction, stroke and heart failure) are presented in Fig. 2A–D. The associated risk of one-year MACE decreased (one-year HR in 2019 vs. 2010; 0.90; 95% CI 0.81–1.00, *p* = 0.045), while the risk of mortality was unaltered (Fig. 3A and B).

### Causes of death after coronary angiography and by year after index coronary angiography

Trends in cause of death the first year after angiography are depicted in Supplemental Fig. 2, with only marginal differences over the years from 2010 to 2021, where ischemic heart disease (IHD) still predominates. The first year after the index coronary angiography, IHD as cause of death, accounted for 62% of all deaths, declining to 37% in the second year in favor of cancer and thereafter declining to reach a percentage of 25% yearly (Supplemental Fig. 3).

### Cumulative event rate of MACE stratified by group of GLD

**Fig. 4:**
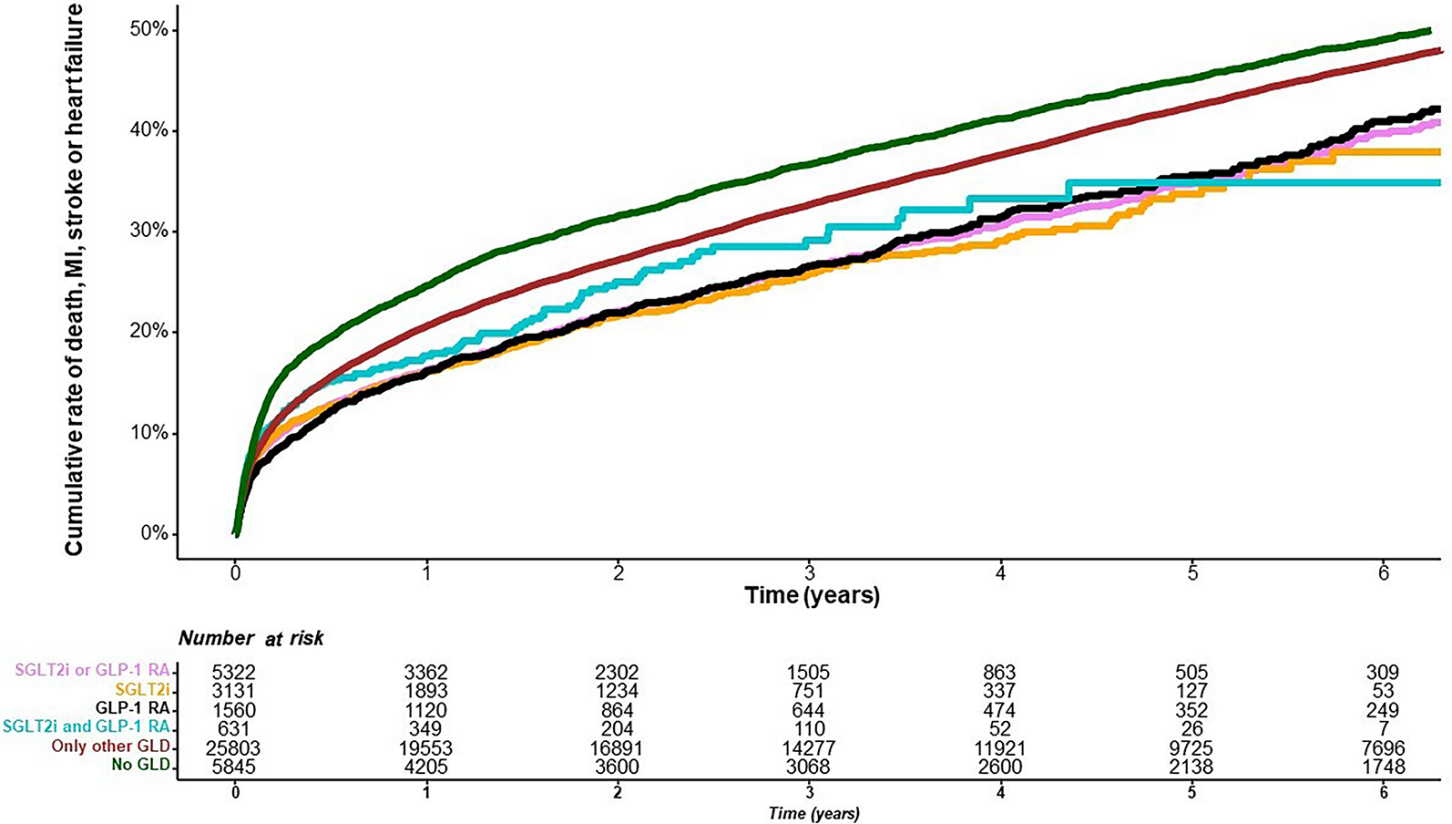
Time to MACE (all-cause death, myocardial infarction, stroke or heart failure) after coronary angiography by classes of glucose-lowering drugs (Green = no GLD, Red = other GLD, Blue = SGLT2i and GLP1-RA, Black = GLP-1 RA, Orange = SGLT2i, Lilac = any of SGLT2i or GLP1-RA)

The cumulative event rate of MACE stratified by GLD group is presented in Fig. 4, with the highest event rate occurring in patients without glucose-lowering treatment (50%) and the lowest rate occurring in patients receiving any of SGLT2i or GLP-1 RA (41%). MACE the first year after the index coronary angiography occurred in 16.0% of patients with any of SGLT2i or GLP-1 RA, 21.1% in patients with other GLD and 39.1% in patients without any GLD. Adjusted associated risk for MACE was highest in those without GLD (the no GLD group; HR 1.42; 95% CI 1.32–1.52, *p* < 0.001), followed by those with treatment with other GLD (HR 1.10; 95% CI 1.03–1.17, *p* = 0.004) when treatment with any of SGLT2i or GLP-1 RA served as a reference (Supplemental Table 2 depicting unadjusted and adjusted figures).

All-cause death within one year occurred in 2.1% of patients with any of SGLT2i or GLP-1 RA, 4.5% in patients with other GLD and 26.4% in patients without any GLD. The cumulative long-term event rate of all-cause death by GLD group is presented in Supplemental Fig. 4, showing the highest mortality in patients without GLD (approximately 31% after six years) and the lowest in patients receiving any of SGLT2i or GLP-1 RA (approximately 16% after six years). The adjusted associated risk of all-cause death was highest in patients with no GLD (HR 2.61; 95% CI 2.32–2.93, *p* < 0.001), followed by those treated with other GLD (HR 1.60; 95% CI 1.43–1.80, *p* < 0.001; Supplemental Table 2), where treatment with any of SGLT2i or GLP-1 RA served as a reference.

## Discussion

In this large, unselected cohort of patients with diabetes and CAD, there are four major findings. First, in Sweden, there has been a change in prescription patterns with the rapid increase in the use of SGLT2i and GLP-1 RA and in accordance with recent guidelines, even if this change is not yet satisfactory. Second, there are signs of ineffective use of these cardioprotective drugs, including inequality favoring the healthiest CVD patients with diabetes and thus a missed opportunity to protect those at high risk of an additional event. Third, we demonstrate encouraging declining trends in the risk of MACE in recent years in patients with diabetes and CAD. Fourth, we show that CVD is still the major cause of mortality, at approximately 60%, in the first years following a coronary event. However, cancer- and endocrinological/diabetes-related complications catch up already the second year and by longer follow-up time even though IHD still accounts for approximately one-quarter of all causes of death.

Landmark trials and fast-updated diabetes guidelines have indeed rapidly affected Swedish prescription patterns in favor of SGLT2i and GLP-1 RA as first line recommendation in CVD, while as for instance elderly drugs without CV-evidence are at second- or third-line recommendation. In 2021, the proportion of patients who were treated with SGLT2i/ GLP-1 RA was as large as that of patients treated with other GLD, approximately 40%; however, a more reasonable proportion could be 80–90%, considering contraindications, comorbidities, and type 1 diabetes. Thus, there is still room for improvement. The landmark trials support that SGLT2i/ GLP-1 RA are effective irrespective of baseline treatment with other GLD and should be used regardless of glucose control [11, 12]. In this real-life analysis, we demonstrate that there seems to be a preferential use of prescribing those cardioprotective drugs to a low-risk rather than to a high-risk patient. It was previously shown that physicians tend to prescribe novel drugs to less vulnerable patients [14] while the absolute effects are often more prominent in high-risk settings. One explanation could be that it is easier and less time-consuming to prescribe a novel drug to a healthier and younger person without previous glucose-lowering agents. Indeed, we found that patients prescribed SGLT2i/ GLP-1 RA were younger, more often drug naïve, more often men and less often had heart failure and other comorbidities and that patients with previous heart failure were left without cardio-protective treatment to a greater extent. Hence, the lower event rate in patients on SGTL2i/ GLP-1 RA in this study can possibly not fully be attributed to the effect of the drugs but rather be explained by the prescription to patients at lower CV risk. Notably, patients included in CV outcome trials (CVOTs) had several years of duration of type 2 diabetes, had high HbA1c levels and often had a combination of GLD [7–9]. In addition, patients with diabetes and a history of previous MI have a high proportion of repeating events as heart failure [15–17]. In our real-world analysis, we found that SGLT2i/ GLP-1 RA were often prescribed to GLD-naïve patients; for example, 17% of those receiving SGLT2i were previously drug naïve (and with an even greater proportion in the years 2020–2021; 20%). The use of SGLT2i/ GLP-1 RA in those who were previously GLD naïve can be explained by the rapidly changing guidelines for the management of diabetes, which recommend these novel cardioprotective GLD for newly discovered diabetes regardless of HbA1c. The conclusions of the guidelines supporting the extended use of SGLT2i/ GLP-1 RA might have overshadowed the effectiveness of SGLT2i/GLP-1 RA in those with signs of polyvascular disease at greater absolute CV risk [18].

There is evidence of an equal cardio-protective effect of SGLT2i/ GLP-1 RA across sexes, and the proportion of women was approximately 40% in CV outcome trials [7–9]. Unfortunately, we found that women were less often prescribed these novel GLD, and this trend was even exaggerated in the latter years (2020–2021) of the present study. One reason for depriving women SGLT2i/ GLP-1 RA could be that women with diabetes and CAD are older and therefore considered to be more vulnerable, again biasing the physician’s judgment. Additionally, it cannot be ruled out that nonretired, well-educated individuals who have better preconditions to discuss their medication are prescribed more modern and costly therapies. Previous studies have demonstrated that gender, education and socioeconomics matter in terms of offered treatment [19–21]. In the US, it was recently shown that more financially advantaged persons with better insurance contracts are prescribed SGLT2i/ GLP-1 RA to a greater extent [22]. Further supporting this inequality use is the report from Stolfo et al. where the novel heart failure SGLT2i class was preferably given to men [23].

This is aggravating if we want to achieve an equalization in prognosis in patients with CAD. Although the analyses in this study were not propensity matched, patients on SGLT2i/ GLP-1 RA had a significantly better prognosis than those on other GLD, an observation that may be explained by novel GLD being prescribed to the healthiest patients.

There are consequences of not prescribing novel cardioprotecting GLD to high-risk diabetes patients. Since mortality, as we demonstrate is mainly driven by IHD the first year, and especially nonfatal event rates such as heart failure are high, there are currently few ways to change this costly hospital-demanding care other than offering at least one of the new cardioprotective GLD. Despite these findings, we observed encouraging trends in CV prognosis over the last few years, with a progressively reduced annual hazard ratio (HR) for major adverse cardiovascular events, particularly in the years 2018–2019 (excluding the years 2020–2021 due to the COVID-19 pandemic). On the contrary, mortality was not reduced over time. We believe this is due to the low absolute mortality rate compared to the much higher MACE rate (especially for heart failure). Also, mortality was to a lower extent than previously explained by cardiovascular causes (approximately 60%), especially for those surviving the first year.

This study indicates that there is indeed a possibility to improve the long-standing plateaued outcome in persons with diabetes and CAD. The mechanisms behind this trend have not been explored in detail in this study; however, it is likely that improved overall risk factor management according to repeated guidelines in the last decade and increased use of the novel cardioprotective GLD, both before and quickly after discharge from the coronary event, has had an impact.

### Strengths and limitations

A major strength of this study is the large unselected nationwide cohort, which included diabetes patients with both acute myocardial infarction and stable CAD, enabling analyses of prescription patterns and prognosis in real life outside the clinical trial setting. A healthy selection bias was limited, as mortality during the first 30 days was excluded from the analysis. Notably, patients with myocardial infarction who did not undergo coronary angiography were excluded from this study. From a prognostic perspective, one limitation is that we did not adjust for other CVD medications since information on these variables was not available for the whole cohort. However, we know from other reports that other secondary preventive medications are given to a high extent [24]. Furthermore, the observational study design does not allow any interpretation of causal relationships between the treatment and the observed outcomes.

As patients were not categorized by type of diabetes, approximately 5% of the patients included probably have type 1 diabetes, based on previous research from the SWEDEHEART registry [3], which could influence the medication strategy in a rather low proportion. Most patients with coronary artery disease and insulin use are type 2 diabetes. Furthermore, there was no information on diabetes duration, diabetes complications and risk of hypoglycemia, all of which could have influenced the choice of GLD. Also, we lack information on HbA1c, which still is an important predictor for cardiovascular outcome [16, 25].

## Conclusion

There is a fast-changing prescription pattern of GLD in patients with type 2 diabetes and CAD in Sweden in accordance with international guidelines, although there is still room for improvement. However, there are worrying signals of inefficient use, with trends to prioritize novel cardioprotective GLD to a population at lower cardiovascular risk and with an associated better outcome, thus missing those at high risk of future events who also should be considered treatment with SGLT2i and GLP-1 RA. Therefore, we would like to increase the clinicians’ awareness of the risk of missing an opportunity to give effective CV-preventive drugs also to diabetes patients with longer duration, higher HbA1c, women and diabetes patients already on multiple diabetes treatments and insulin. Encouragingly, a trend was observed with improved MACE, beginning in 2015, and with less mortality from cardiovascular causes.

### Supplementary Information

Supplementary Material 1.

## Data Availability

The data underlying this article are available in the article and in its online supplementary material.

## Abbreviations

AMI: Acute myocardial infarction
CAD: Coronary artery disease
CI: Confidence interval
CV: Cardiovascular
CVD: Cardiovascular disease
GLD: Glucose-lowering drugs
GLP-1 RA: Glucagon Like Peptide Receptor Agonists
HF: Heart failure
HR: Hazard ratio
ICD-code: International Statistical Classification of Diseases and Related Health Problems
IHD: Ischemic heart disease
LVEF: Left ventricular ejection fraction
MACE: Major adverse cardiovascular events
MI: Myocardial infarction
NPR: National Patient Registry
NSTEMI: non-ST-segment elevation myocardial infarction
PDR: Prescribed Drug Registry
SCAAR: Swedish angiography and angioplasty registry
SGLT2i: Sodium Glucose Lowering Transport 2 receptor inhibitors
STEMI: ST-segment elevation myocardial infarction
