# Supplementary material for: Trends in prognosis and use of SGLT2i and GLP-1 RA in patients with diabetes and coronary artery disease
Source: Cardiovasc Diabetol. 2024 Aug 7;23:290. doi: 10.1186/s12933-024-02365-1 (PMC11304712; doi:10.1186/s12933-024-02365-1)
Supplement: Supplementary file 1 — Supplementary Material 1. [file 12933_2024_2365_MOESM1_ESM.docx]

**Supplemental material**

**Tables**

**Table 1.** Patient characteristics at baseline in 2020-2021, mean (SD) or n (%) stratified by glucose-lowering drugs (GLD).

p value 1= p value across the following groups: SGLT2i, GLP1-RA, SGLT2i and GLP1-RA, Other GLD and No GLD.

p value 2= p value across the following groups: Any of SGLT2i or GLP1-RA and Other GLD

|  | **SGLT2i**  **(n=1655)** | **GLP1-RA**  **(n=483)** | **SGLT2i + GLP1-RA**  **(n=378)** | **Other GLD**  **(n=2671)** | **No GLD (n=879)** | **p value 1** | **Any SGLT2i/GLP1-RA**  **(n=2516)** | **p value 2**  **Any**  **SGLT2i/GLP1-RA**  **vs. Other GLD** |
| --- | --- | --- | --- | --- | --- | --- | --- | --- |
| Age (years) | 67.2 (10.5) | 67.0 (10.3) | 63.5 (9.4) | 70.0 (10.6) | 73.2 (10.7) | <0.001 | 66.6 (10.4) | <0.001 |
| Sex (female) | 403 (24.4) | 152 (31.5) | 87 (23.0) | 902 (33.8) | 326 (37.1) | <0.001 | 642 (25.5) | <0.001 |
| Current smoker | 301 (18.2) | 69 (14.3) | 81 (21.4) | 384 (14.4) | 128 (14.6) | <0.001 | 451 (17.9) | 0.004 |
| BMI (kg/m^2^) | 29.2 (5.2) | 31.8 (5.6) | 30.9 (5.2) | 28.8 (5.3) | 28.2 (7.4) | <0.001 | 29.9 (5.4) | <0.001 |
| Weight (kg) | 87.5 (17.1) | 95.3 (18.5) | 94.2 (18.2) | 85.1 (17.7) | 82.1 (17.8) | <0.001 | 90.0 (17.9) | <0.001 |
| **Left Ventricular Ejection fraction (LVEF %)** |  |  |  |  |  | <0.001 |  | <0.001 |
| LVEF ≥50% | 657 (52.4) | 174 (64.0) | 133 (52.0) | 1046 (63.9) | 269 (45.4) |  | 964 (54.1) |  |
| LVEF 40-49% | 313 (25.0) | 55 (20.2) | 71 (27.7) | 348 (21.3) | 125 (21.1) |  | 439 (24.6) |  |
| LVEF 30-39% | 199 (15.9) | 32 (11.8) | 40 (15.6) | 173 (10.6) | 99 (16.7) |  | 271 (15.2) |  |
| LVEF <30% | 85 (6.8) | 11 (4.0) | 12 (4.7) | 70 (4.3) | 99 (16.7) |  | 108 (6.1) |  |
| Blood glucose (mmol/L) | 11.7 (5.0) | 12.3 (5.2) | 13.1 (5.5) | 11.0 (5.1) | 10.7 (5.9) | <0.001 | 12.0 (5.1) | <0.001 |
| CRP (mg/L) | 18.1 (41.4) | 18.0 (39.0) | 16.8 (43.6) | 15.2 (33.1) | 26.0 (50.6) | <0.001 | 17.9 (41.3) | 0.050 |
| Creatinine (mmol/L) | 81.1 (31.4) | 91.9 (54.6) | 83.6 (52.2) | 98.3 (85.0) | 114.7 (108.0) | <0.001 | 83.5 (40.5) | <0.001 |
| **Previous disease** |  |  |  |  |  |  |  |  |
| Heart failure | 78 (4.7) | 31 (6.4) | 15 (4.0) | 161 (6.0) | 89 (10.1) | <0.001 | 124 (4.9) | 0.094 |
| PAD | 52 (3.1) | 28 (5.8) | 18 (4.8) | 149 (5.6) | 76 (8.6) | <0.001 | 98 (3.9) | 0.005 |
| MI | 135 (8.3) | 31 (6.5) | 27 (7.3) | 176 (6.7) | 71 (8.3) | 0.243 | 193 (7.8) | 0.139 |
| Stroke | 106 (6.4) | 42 (8.7) | 27 (7.1) | 209 (7.8) | 98 (11.1) | 0.001 | 175 (7.0) | 0.253 |
| Renal failure | 51 (3.1) | 40 (8.3) | 20 (5.3) | 228 (8.5) | 98 (11.1) | <0.001 | 111 (4.4) | <0.001 |
| Cancer | 46 (2.8) | 12 (2.5) | 5 (1.3) | 102 (3.8) | 33 (3.8) | 0.041 | 63 (2.5) | 0.009 |
| Dementia | 7 (0.4) | 0 (0) | 0 (0) | 13 (0.5) | 12 (1.4) | 0.002 | 7 (0.3) | 0.324 |
| Hypertension | 1202 (73.3) | 403 (883.4) | 304 (81.9) | 2134 (80.6) | 674 (78.4) | <0.001 | 1909 (76.5) | <0.001 |
| Hyperlipidemia | 890 (54.8) | 299 (62.2) | 221 (59.6) | 1646 (62.4) | 414 (48.5) | <0.001 | 1410 (56.9) | <0.001 |
| **Indication** |  |  |  |  |  | <0.001 |  | <0.001 |
| Stable CAD | 352 (21.3) | 193 (40.0) | 113 (29.9) | 936 (35.0) | 194 (22.1) |  | 658 (26.2) |  |
| NSTEMI | 706 (42.7) | 196 (40.6) | 146 (38.6) | 1136 (42.5) | 365 (41.5) |  | 1048 (41.7) |  |
| STEMI | 597 (36.1) | 94 (19.5) | 119 (31.5) | 599 (22.4) | 320 (36.4) |  | 810 (32.2) |  |
| **Angiographic findings** |  |  |  |  |  | <0.001 |  | <0.001 |
| Normal | 152 (9.2) | 98 (20.3) | 42 (11.1) | 463 (17.3) | 118 (13.4) |  | 292 (11.6) |  |
| 1-vessel | 579 (35.0) | 154 (31.9) | 151 (39.9) | 870 (32.6) | 249 (28.3) |  | 884 (35.1) |  |
| 2-vessel | 412 (24.9) | 112 (23.2) | 87 (23.0) | 593 (22.2) | 216 (24.6) |  | 611 (24.3) |  |
| 3-vessel | 380 (23.0) | 84 (17.4) | 78 (20.6) | 517 (19.4) | 194 (22.1) |  | 542 (21.5) |  |
| Left main | 132 (8.0) | 33 (6.8) | 19 (5.0) | 225 (8.4) | 102 (11.6) |  | 184 (7.3) |  |
| **Revascularization method** |  |  |  |  |  |  |  |  |
| PCI | 1253 (75.7) | 329 (68.1) | 294 (77.8) | 1781 (66.7) | 623 (70.9) | <0.001 | 1876 (74.6) | <0.001 |
| CABG within 3 months  after index angiography | 84 (5.1) | 25 (5.2) | 21 (5.6) | 140 (5.2) | 19 (2.2) | 0.004 | 130 (5.2) | 0.954 |
| Stent during PCI (n) | 1.4 (1.0) | 1.4 (1.1) | 1.3 (1.0) | 1.3 (1.1) | 1.4 (1.1) | 0.521 | 1.4 (1.0) | 0.280 |
| **Medications within 6 months to index angiography** |  |  |  |  |  |  |  |  |
| Insulin | 492 (29.7) | 228 (47.2) | 193 (51.1) | 1095 (41.0) | 0 (0) | <0.001 | 913 (36.3) | 0.001 |
| Metformin | 1208 (73.0) | 303 (62.7) | 285 (75.4) | 1764 (66.0) | 0 (0) | <0.001 | 1796 (71.4) | <0.001 |
| Sulfonylurea | 81 (4.9) | 22 (4.6) | 21 (5.6) | 116 (4.3) | 0 (0) | <0.001 | 124 (4.9) | 0.349 |
| SGLT2i | 1655 (100) | 0 (0) | 378 (100) | 0 (0) | 0 (0) | <0.001 | 2033 (80.8) | <0.001 |
| DPP4i | 234 (14.1) | 36 (7.5) | 29 (7.7) | 468 (17.5) | 0 (0) | <0.001 | 299 (11.9) | <0.001 |
| GLP-1 RA | 0 (0) | 483 (100) | 378 (100) | 0 (0) | 0 (0) | <0.001 | 861 (34.2) | <0.001 |
| **Any diabetes medication**  **prior admission** | 1326 (80.1) | 446 (92.3) | 349 (92.3) | 2341 (87.6) | 361 (41.1) | <0.001 | 2121 (84.3) | 0.001 |

SGLT2i: Sodium Glucose Lowering Transport 2 receptor inhibitors. GLP-1 RA: Glucagon Like Peptide Receptor Agonists. BMI: body mass index. CRP: C-reactive protein. PAD: peripheral artery disease. MI: myocardial infarction. CAD: Coronary artery disease. NSTEMI: non-ST-segment elevation myocardial infarction. STEMI: ST-segment elevation myocardial infarction. PCI: percutaneous coronary intervention. CABG: coronary artery bypass graft. DPP4i: Dipeptidyl peptidase 4 inhibitors.

**Table 2**. Unadjusted and adjusted risk (HR CI 95%) for MACE and all-cause death after index coronary angiography in patients stratified by glucose-lowering drugs (GLD). Patients treated with any SGLT2i or GLP1-RA served as a reference group with an HR of 1.0.

| **Event** | **Unadjusted**  **HR (95% CI)** | **p value** | **Adjusted***  **HR (95%CI)** | **p value** |
| --- | --- | --- | --- | --- |
| *MACE* |  |  |  |  |
| Any SGLT2i or GLP-1 RA | 1 (referent) |  | 1 (referent) |  |
| Other GLD | 1.33 (1.26-1.41) | <0.001 | 1.10 (1.03-1.17) | 0.004 |
| No GLD | 2.08 (1.96-2.21) | <0.001 | 1.42 (1.32-1.52) | <0.001 |
|  |  |  |  |  |
|  |  |  |  |  |
| *All-cause death* |  |  |  |  |
| Any SGLT2i or GLP-1 RA | 1 (referent) |  | 1 (referent) |  |
| Other GLD | 2.14 (1.92-2.38) | <0.001 | 1.60 (1.43-1.80) | <0.001 |
| No GLD | 4.53 (4.06-5.06) | <0.001 | 2.61 (2.32-2.93) | <0.001 |

***** Adjusted for age, sex, smoking, previous diagnosis of MI/ heart failure/ cancer/ hypertension/ hyperlipidemia/ renal failure/ stroke/ peripheral artery disease, year, indication, and angiographic findings. MACE= Major adverse cardiovascular event (all-cause death, myocardial infarction, stroke or heart failure). GLD= Glucose lowering drugs.

**Figure 1.** Use of other GLD by treatment group.


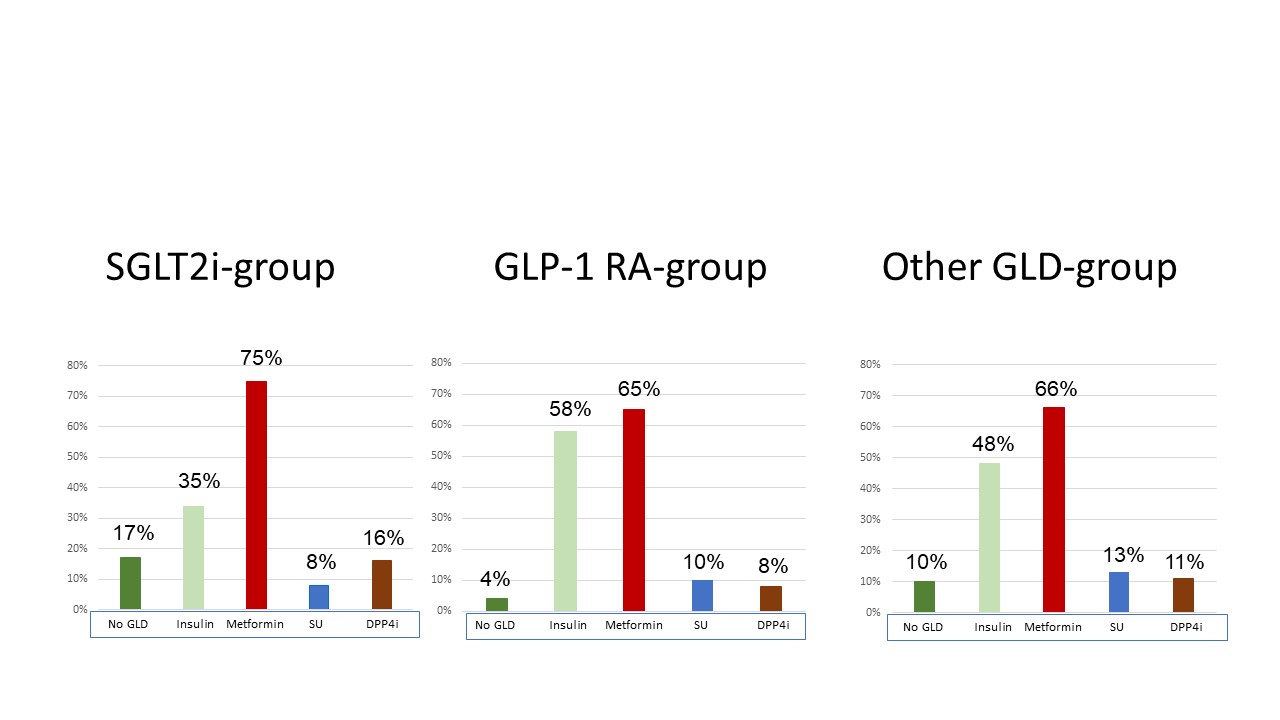


SGLT2i: Sodium Glucose Lowering Transport 2 receptor inhibitors. GLP-1 RA: Glucagon Like Peptide Receptor Agonists. GLD: Glucose-lowering drugs. SU: Sulfonylureas. DPP4i: Dipeptidyl peptidase 4 inhibitors.

**Figure 2.** Causes of death in diabetes patients the first year after coronary angiography each year from 2010 to 2021.


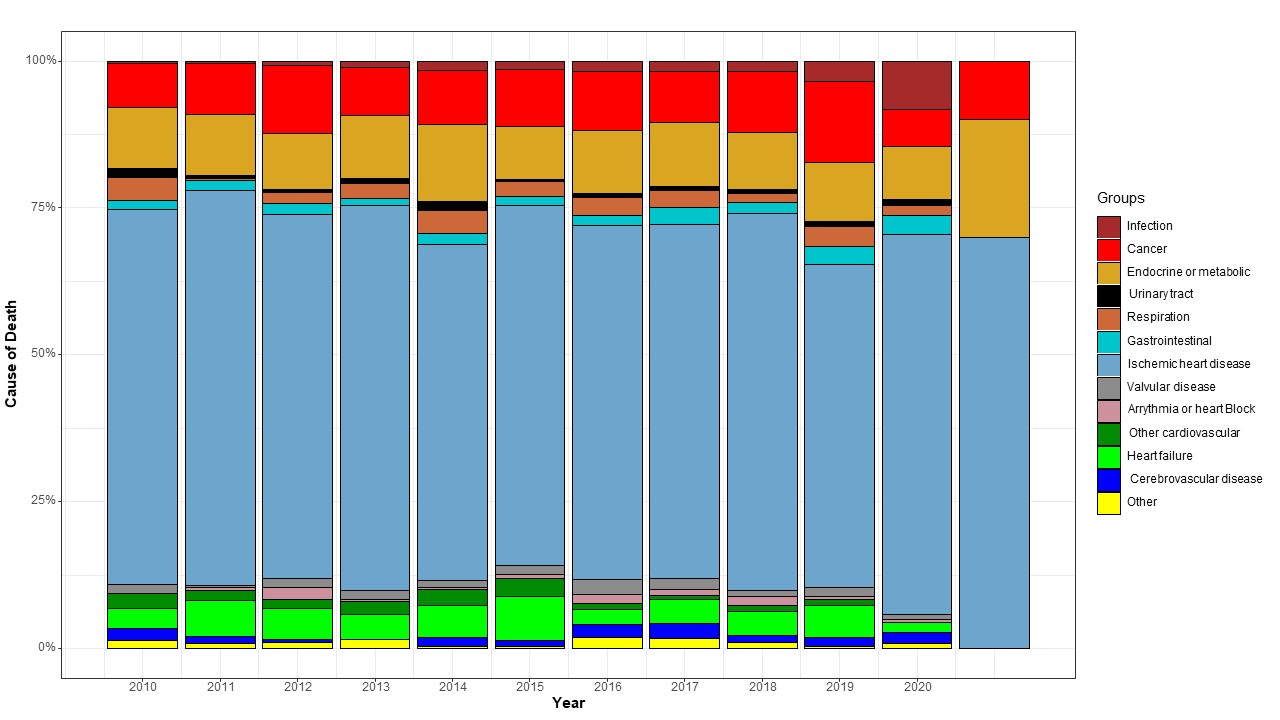


**Figure 3.** Trends in cause of death in diabetes patients by year after first coronary angiography showing ischemic heart disease as a dominant cause of death in the first year and thereafter declining.


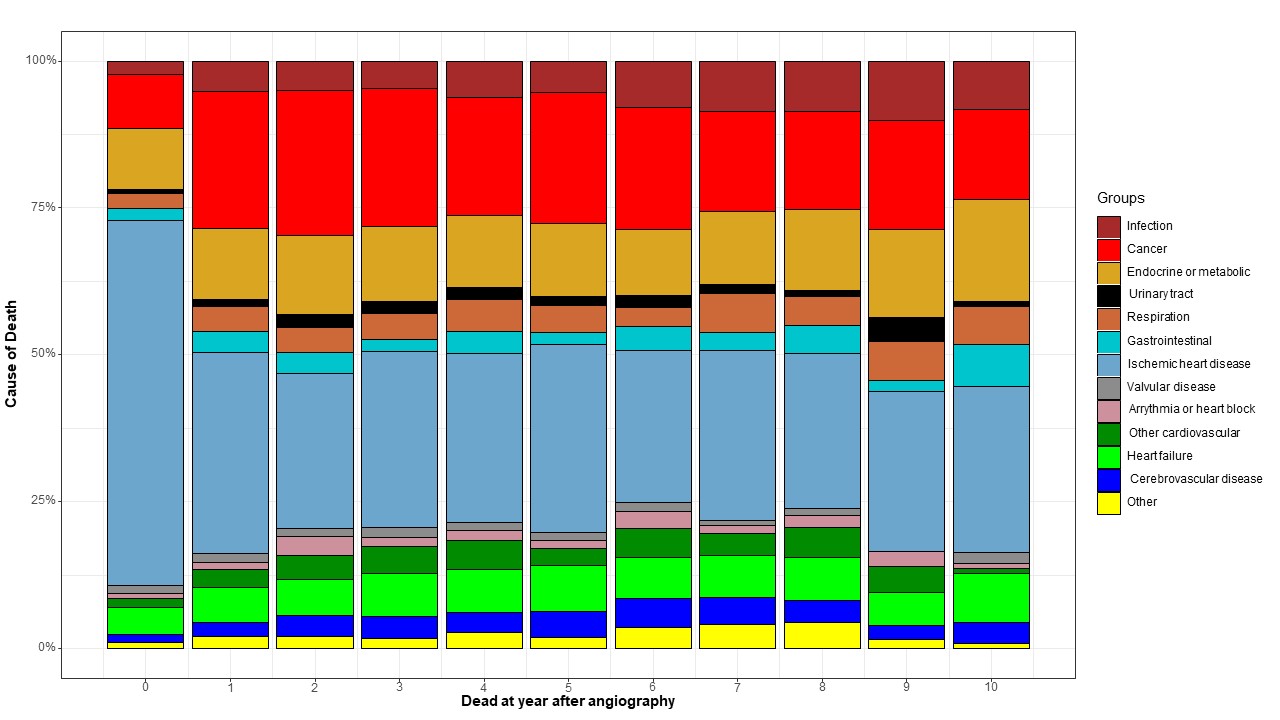


**Figure 4.** Time to all-cause death after coronary angiography by classes of glucose-lowering drugs. (Green= no GLD, Red= other GLD, Blue= SGLT2i and GLP1-RA, Black= GLP-1 RA, Orange= SGLT2i, Lilac= any of SGLT2i or GLP1-RA).

**
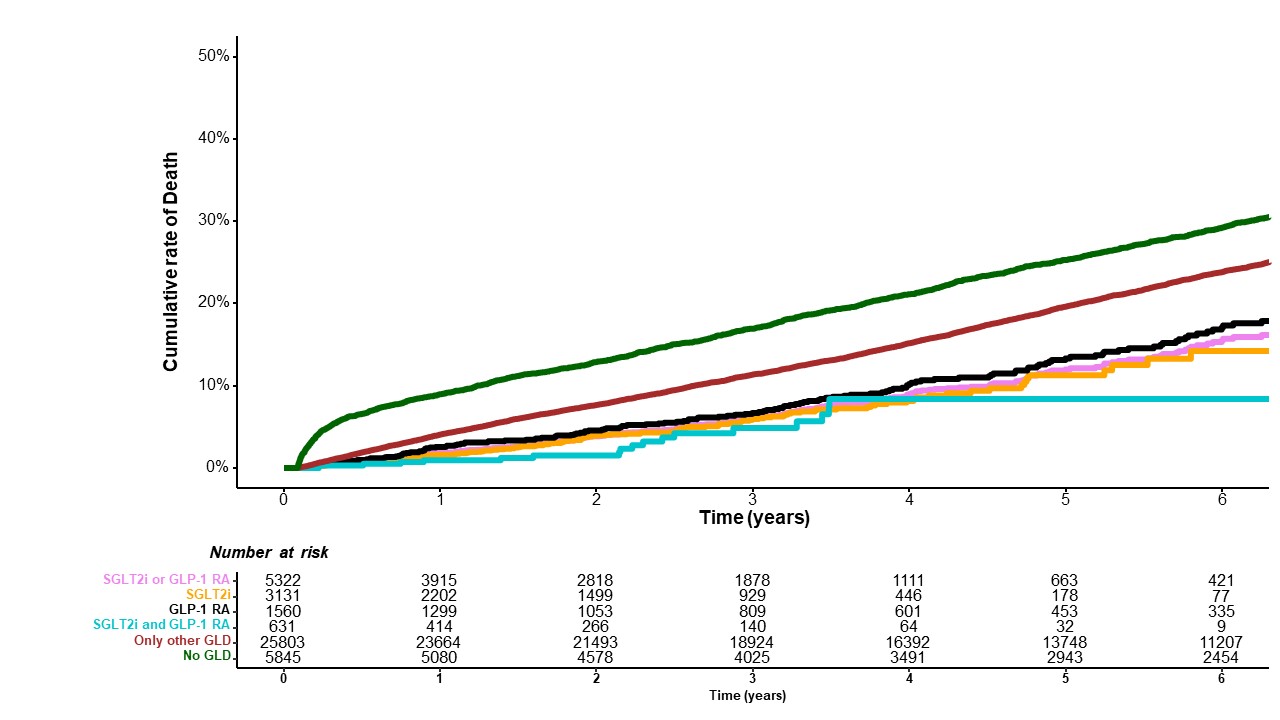
**

SGLT2i: Sodium Glucose Lowering Transport 2 receptor inhibitors. GLP-1 RA: Glucagon Like Peptide Receptor Agonists. GLD: Glucose-lowering drugs.
